# X-Linked Retinoschisis: Phenotypic Variability in a Chinese Family

**DOI:** 10.1038/srep20118

**Published:** 2016-01-29

**Authors:** Yangyan Xiao, Xiao Liu, Luosheng Tang, Xia Wang, Terry Coursy, Xiaojian Guo, Zhuo Li

**Affiliations:** 1 Department of Ophthalmology, Second Xiangya Hospital, Central South University, Changsha, Hunan, China; 2 Human Genome Sequencing Center, Baylor College of Medicine, Houston, Texas, USA; 3 Cullen eye institute, Baylor College of Medicine, Houston, Texas, USA

## Abstract

X-linked juvenile retinoschisis (XLRS), a leading cause of juvenile macular degeneration, is characterized by a spoke-wheel pattern in the macular region of the retina and splitting of the neurosensory retina. Our study is to describe the clinical characteristics of a four generations of this family (a total of 18 members)with X-linked retinoschisis (XLRS) and detected a novel mutations of c.3G > A (p.M1?) in the initiation codon of the RS1 gene. by direct sequencing.Identification of this mutation in this family provides evidence about potential genetic or environmental factors on its phenotypic variance, as patients presented with different phenotypes regardless of having the same mutation. Importantly, OCT has proven vital for XLRS diagnosis in children.

X-linked juvenile retinoschisis (XLRS, MIM 312700), first described in 1898 by German ophthalmologist Josef Haas, is a relatively common retinal degenerative disease that affects male patients early in life. XLRS affects approximately 1 in 5,000–25,000 men^1^,^2^. XLRS is caused by mutations in the RS1 gene in Xp22.2 and was identified by positional cloning in 1997^2^. Human RS1 contains six separate exons and encodes for the 224 amino acid cell-surface protein retinoschisin (RS1)^3^,^4^. RS1 is highly expressed by retinal photoreceptors and bipolar cells and interacts with the surfaces of these cell types to stabilize the organization of the retina^4^,^5^,^6^.

Patients with severe cases and older individuals display atrophic macular in the retinal pigment epithelium^7^.White flecks and dots, and fundus albipunctatus are associated with the severe foveal and peripheral retinoschisis^7^. Complications include vitreous hemorrhage, choroidal sclerosis, retinal detachment, and neovascular glaucoma, and often leads to a poor prognosis^8^. Electroretinogram (ERG) can be used for the clinical diagnosis of XLRS. Because the main functional defect may occur after photo transduction, the full-field ERG in patients with XLRS exhibits a relative preservation of an a-wave amplitude which indicate the function of photo transduction. But they have a substantial reduction of the dark-adapted b-wave amplitude originating in inner retinal cell activity^9^. Because XLRS patients with *RS1* mutations may have normal ERG, the diagnosis of XLRS cannot be based on ERG alone^10^. Retinal fundus examination, optical coherence tomography (OCT), microperimeter, and fundus fluorescein angiography (FFA), as well as *RS1* mutational analysis, are increasingly used in its diagnosis^10^.

In this study, we report finding in a Chinese family with XLRS. We identified a novel mutation, c.3G > A (p.M1?), in the RS1 gene. This mutation co-segregates with disease in the family and likely results in loss of RS1protein production. This mutation expands the mutational spectrum of XLRS and the phenotypes of the affected members of this family provide evidence of potential genetic or environmental factors on XLRS prognosis.

## Methods

### Clinical examination

The clinical protocol was approved by the Second Xiangya Hospital Institutional Review Board, and the tenets of the Declaration of Helsinki were followed. Informed, written consent was obtained before blood sample acquisition. Eighteen members of the family were enrolled in the study. All the individuals underwent standard ophthalmic examinations including evaluation of best-corrected visual acuity (BCVA), non-contacted tonometry, slit-lamp biomicroscopic examination, computerized perimetry (Oculus Twinfield). After pupil dialation with tropicamide (1%) and phenylephrine hydrochloride (2.5%), patients were examined by indirect ophthalmoscopy, OCT (Zeiss Cirrus HD OCT –2000, Carl Zeiss meditec, Inc. Dublin, CA; Spectralis, Heidelberg, Germany), fundus photography (TOPCON TRC 50 DX). Some patients received intravenous fluorescein angiography (FA. Spectralis, Heidelberg, Germany) and Goldman applanation tonometry. ERG (Espion V5, Diagnosys LLC, MA, USA) was performed according to standard testing protocols recommended by the International Society for Clinical Electrophysiology of Vision (ISCEV). Diagnosis of XLRS was based upon history, clinical examination, and electroretinogram findings when available.

### Polymerase chain reaction and molecular genetic studies

Genomic DNA was isolated from peripheral leukocytes using a QIAamp DNA Blood Mini Kit (Qiagen, Hilden, Germany) according to the manufacturers’ protocol. Mutational screening of each coding exon was performed using the primer sequences that were previously published^11^. After purification, amplicons were sequenced using on an ABI 3730 Genetic Analyzer (ABI, Foster City, CA, USA) at the Beijing Institute of Genomics (Shenzhen, China). Mutations were identified by comparing coding sequences with those of 100 normal X chromosomes and unaffected family members. Sequences were assembled and analyzed with Lasergene SeqMan software (DNASTAR, Madison, WI, USA). The results were compared with an *RS1* reference sequence (GeneBank accession number: NM_000330). Mutations were annotated following the nomenclature recommended by the Human Genome Variation Society (HGVS).

## Results

### Clinical Findings

**Figure 1:**
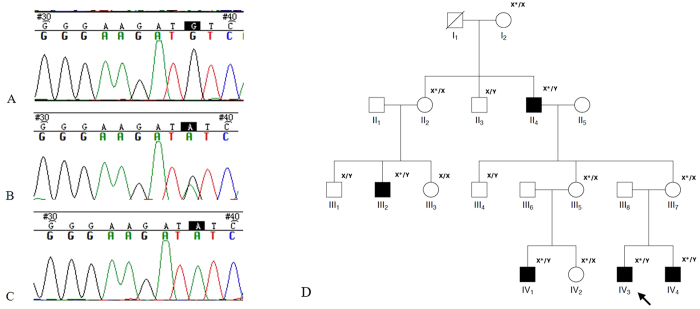
Pedigree of the family in this study and part of DNA sequencing. (**A**) Normal *RS1* gene (III-3); (**B**) Heterozygous female carrier (III-7); (**C**) Male patient (III-4); (**D**) The corresponding genotypes depicted next to each sequenced individuals in this study. (X* means the X chromosome with mutation, hence X*/Y means effected male).

**Figure 2:**
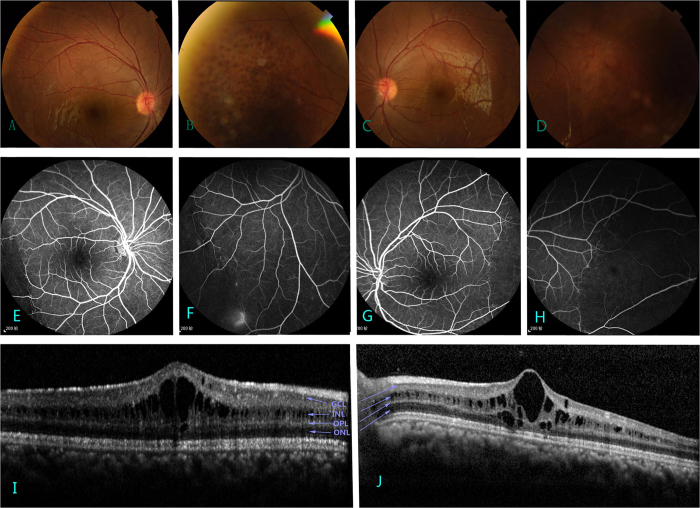
Images of the proband (IV-3). Panels (**A**–**D**) show grayish pigmentation in temporal quadrant both eyes. Schisis is only observed under fudusscopic examination; (**E**–**H**) FA present no infusion in peripheral retina, with little inferotemporal linkage in right eye and no macula linkage in both eyes; (**I**,**J**) The thickness of macular retina is 540 μm in the left eye and 580 μm in right eye determined by OCT. GCL: ganglion cell layer; INL: inner nuclear layer; OPL: outer plexiform layer; ONL: outer nuclear layer.

Three juvenile and two adult patients were affected in this family (Fig. 1d, Table 1), and none had a history of premature delivery. Panel D in Fig. 1 shows the proband (IV-3) of a 12-year-old male suspected to have retinoschisis when decreased vision occurred four years earlier. Best-corrected visual acuity was 20/50 in the right eye (OD) and 20/40 in the left eye (OS). Patient had normal intraocular pressure and slit-lamp biomicroscopy finding in both eyes. Under funduscopic examination, bullous retinoschisis was present in temporal and foveal regions in both eyes, which was confirmed by OCT (Fig. 2i,j). Full-field ERGs demonstrated borderline reduction in the rod-specific ERG, normal maximal ERG a-waves but mild electronegative waveforms. Photopic 30 Hz flicker ERGs were slightly delayed without significant amplitude reduction; transient photopic ERG showed a low b/a ratio 0.56 OD and 0.64 OS.

**Figure 3:**
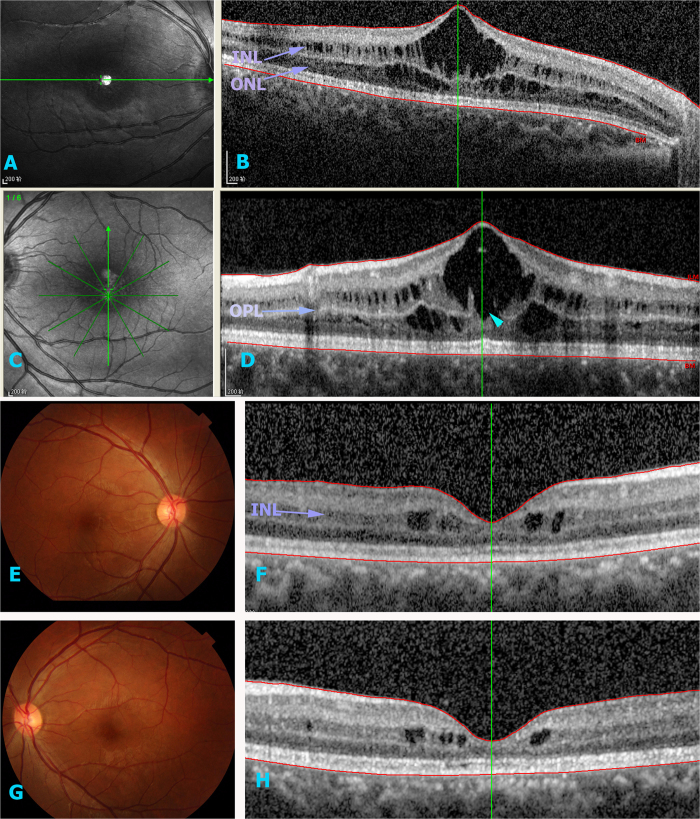
OCT and fundus image of IV4 and III2. (**A**–**D**) depict cystic changes in the outer and inner nuclear layers in IV4. (D) The large cyst effected the outer flexiform layer (arrow). (**E**–**H**) show that III2 contained smaller cysts in the inner nuclear layer of both eyes.

**Figure 4:**
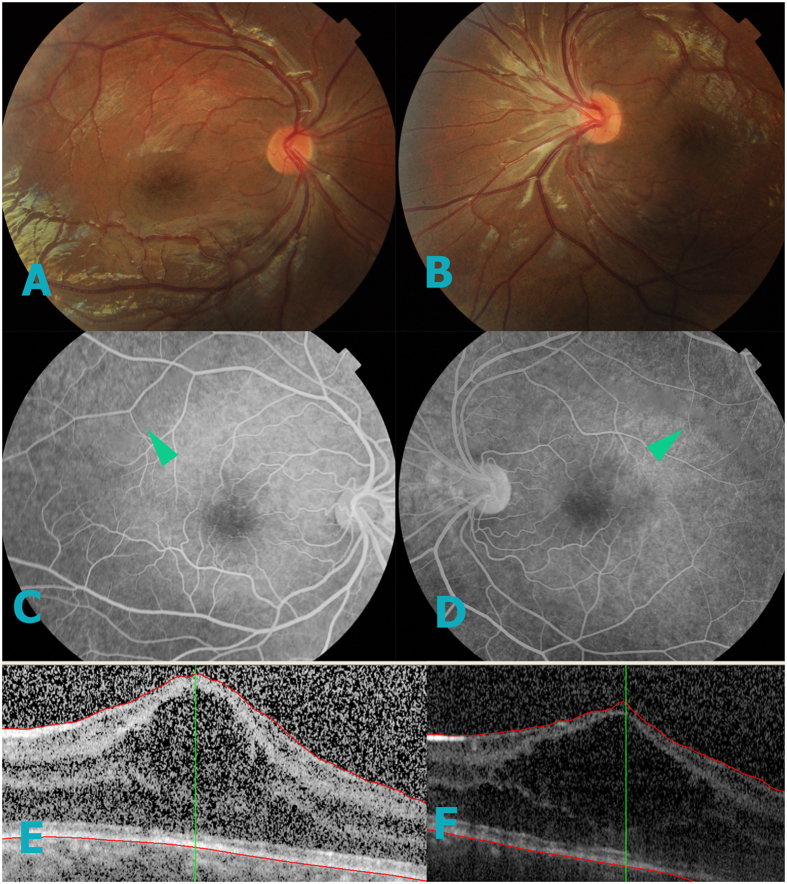
Images of IV-1. (**A**–**D**) There is float (green arrow) and no linkage in FA. (**E**–**F**) OCT results revealed the macula thickness is 651 um in right eye and 548 in left eye.

The patient’s five year old brother (IV-4) was uncooperative and visual acuity measurements were not obtained. However, OCT revealed schisis existing in both the outer and inner nuclear layers (Fig. 3a–d). A six-year-old male (IV-1) was found to have low visual acuity and BCVA were 20/63 OD and 20/160 OS. Slit-lamp biomicroscopy revealed a normal anterior segment. Funduscopic examination presented a spoke-wheel pattern of fovea retinoschisis, which was confirmed by OCT results (Fig. 4). In addition, the periphery schisis had no linkage in FA (Fig. 4). Female siblings were normal in all measurements (III3, III5, III8 and IV-2).

**Figure 5:**
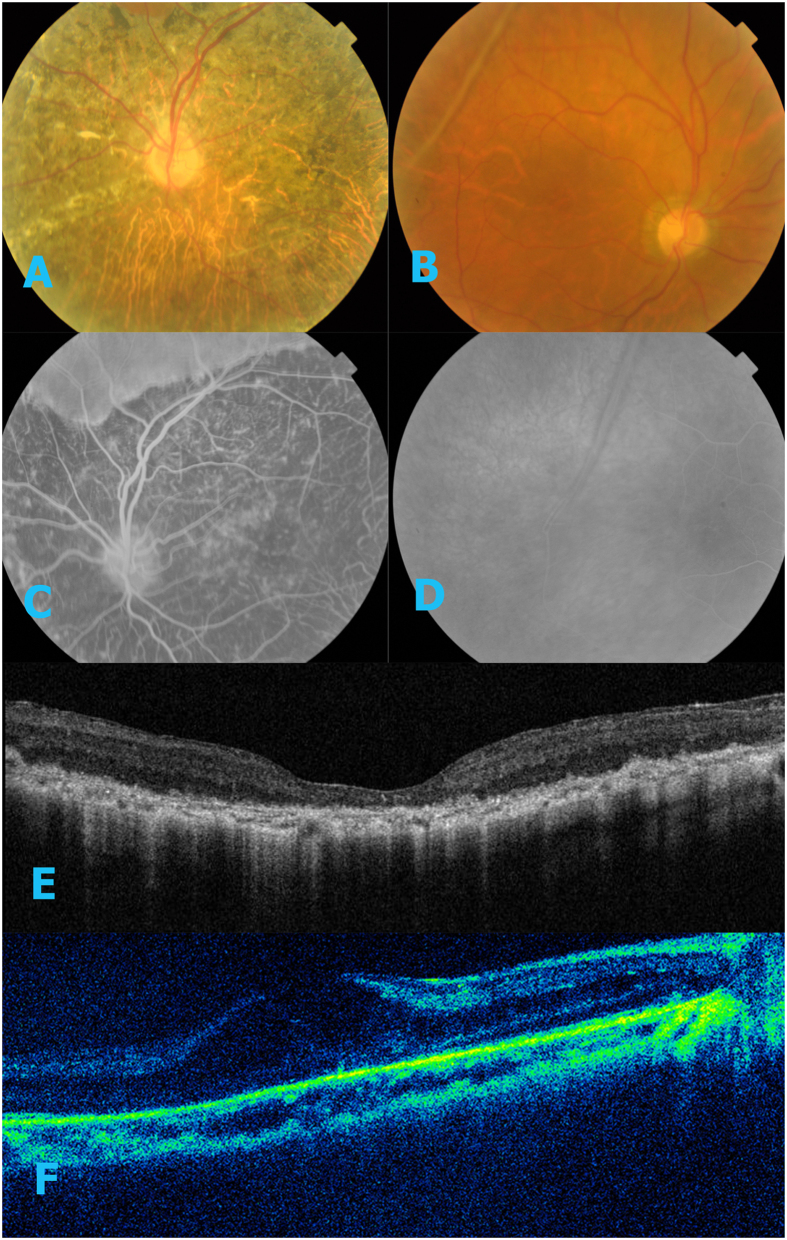
OCT and fundus image of II4. Fundus images of patient II-4 show pigment changes with RPE atrophy in right eye and favela schisis with peripheral schisis in left eye. (**E**,**F**) OCT results showed 82 μm macular retina in the left eye, however the right eye was 340 μm. The OCT results had low quality due to the presence of cataracts, thus a representative image is shown.

An example of an affected adult is the 61-year-old grandfather (II-4, Fig. 5), who has records starting age 50. Visual acuity was 20/200 in the right eye and 20/400 in the left eye with normal intraocular pressure. Patient had no history of hypertension or diabetes mellitus. Slit-lamp biomicroscopy revealed moderate nuclear sclerotic, cortical changes of the lens, and bullous retinoschisis was present in right eye inferotemporal and foveal regions(Fig. 5b,f). The left eye presented no retinoschisis, but had pigmentary abnormalities in the peripheral retina(Fig. 5a).

All affected males had severe retinoschisis, except the patient’s uncle (III-2). III-2 complained of uncorrected vision acuity and OCT showed mild cycstic in macula, which was easily could be missed by the fundus examination. (Fig. 3e–h).

### Molecular Genetic Findings

Direct Sanger sequencing identified a novel hemizygous mutation, c.3G > A (p.M1), in the RS1 gene in the proband (IV3). Our results indicate that this mutation is likely to be the disease-causing mutation. First, further Sanger sequencing of all available family members suggests that this mutation co-segregates with the disease in the family, given that all the affected males are hemizygous, and all the unaffected individuals are either heterozygous (female carriers) or wild type for this mutation (Fig. 1a–c). Second, mutations affecting the same initiation codon p.M1 have been extensively reported in XLRS patients^3^,^12^,^13^,^14^. Third, this methionine is well conserved across different vertebrate species, suggesting functional importance. Lastly, this mutation is likely to cause no protein production since other ATG sequences in the RS1 gene are not likely to be used as the alternative initiation codon (see discussion). Overall these results suggest that this mutation is the underlying cause of XLRS in this family.

## Discussion

XLRS is the leading cause of juvenile macular degeneration in males. Different phenotypes were present in this family, such as one individual with asymmetry clinical symptoms and another with pigmentation. However, all had the same point mutation c.3G > A (p.M1), at the initiation codon of the XLRS1 gene, resulting in a change from an ATG codon to an ATA codon (from Met to Ile). In humans, ATG (methionine), as an initiation codon, signals the initiation of translation. Therefore, its abolishment may result in a dramatically truncated protein or loss of protein production. There are 11 other ATG sequences, including this one, throughout the XLRS1 coding region. Nine of them, which are not in frame, are at the following positions: 47 to 49 base pairs (bp), 80 to 81 bp, 111 to 113 bp, 140 to 142 bp, 186 to 188 bp, 266 to 268 bp, 434 to436 bp, 473 to 475 bp, and 530 to 532 bp. Two sequences in frame (442 to 444 bp and 640 to 642 bp) result in Met148 and Met214. However, both Met148 and Met214 are not imbedded in a proper Kozak sequence to facilitate the initiation of translation. Therefore, due to the lack of this proper ribosomal binding sequence, this mutation is likely to result in loss of protein production. Three other initiation codon mutations (1 A > G 1 A > T and 2 T > C) in the RS1 gene have been previously reported^14^,^15^,^16^.

Over the last 16 years, significant progress has been made in understanding XLRS at the clinical, genetic, molecular, and cellular levels. To date 196 different mutations in the RS1 gene are known to cause XLRS (Leiden Open Variation Database, LOVD version 2.0, Build 31; http://grenada.lumc.nl/LOVD2/eye/home.php?select_db=RS1). The RS1 gene spanning 32.4 kb of genomic DNA is organized in six exons and five intervening regions. Retinoschisin (RS1) protein, expressed and localized in the rodent and human retina^8^,^17^,^18^ and pineal gland^18^, was specifically found at the extracellular surfaces of the inner segments of rod and cone photoreceptors (ONL, outer nuclear layer), most bipolar cells (INL, inner nuclear layer), as well as the two plexiform layers (OPL, outer plexiform layer; IPL, inner plexiform layer). RS1 is organized with four distinct regions: a 23-amino-acid N-terminal signature characteristic for cellular proteins processed through the secretory pathway^2^,^19^, a dominant 157 amino acid discoidin domain (DS domain), a 39 amino acid RS1 domain upstream of the discoidin domain, and a 5 amino acid C-terminal segment. It is thought that the DS domain of RS1 anchors to the surface of photoreceptors and bipolar cells^18^. The whole protein is proposed to be the cell adhesion protein which maintains the cellular organization of the retina or may regulate the fluid balance between the intracellular and extracellular environment through its interactions with a Na/K ATPase-SARM1 complex^6^. Thus the lack of functional RS1 may cause fluid accumulation in the extracellular environment forming a fluid-filled cystic cavity that is observed in OCT and histology, particularly within the photoreceptor and bipolar cell layers (INL and ONL)^20^. The cystic cavities could in turn disrupt the organization of retinal layers resulting in a dysfunction of the photoreceptor-bipolar synapse (OPL, IV4).

The penetrance of XLRS is almost complete but its expressivity is highly variable^8^. First, different mutations are associated with different severity. For example, it has been reported that deletion in exon 1 causes more attenuated ERG, whereas missense mutations are associated with a more benign, slowly progressive disease course^11^,^21^. Second, the same mutation in RS1 can be associated with different clinical presentations, which is the case for our patients^8^,^22^. Comparing his uncle, the proband and his brother had much more severe presentation. What’s more, they also had different phenotypes, such as the pigmentation only happened in their grandfather. One possible explanation is that there are genetic modifiers. Indeed, a quantitative trait locus (mapped on chromosome (Chr) 7, named modifier of Rs1 1 (Mor1) has been found to be responsible for the phenotypic variation in the XLRS mouse model^23^. In another study, Shastry *et al.* found that the common polymorphisms of the CFH, LOC 387715/ARMS2 and HTRA1 genes do not contribute to the phenotypic variability of the XLRS disorder^24^.

Environmental factors may contribute to the phenotypic variability of XLRS. Although there are some reports about uneven progress of the both eyes, most of them are due to complications, such as vitreous hemorrhage and retinal detachment. Our patients had none of them, and it seems he got two different diseases in both eyes (II4). In another family with initiation code mutation, inconformity of phenotype in both eyes has also been reported^14^. Two of the family members had a history of “trauma” and one had ocular surgery and the other developed retinal detachment. Neovasculation and vitreous hemorrhage occurred in another patient with diabetes mellitus. Hence the patients’ experience also play some kind of effect on disease’s progression.(Thus the patient’s medical history also plays an important role in disease progression).

In the past, ERG was used to differentiate the XLRS from other diseases. However, with development in ophthalmic technologies, spectral domain OCT (SD-OCT) is an important diagnostic tool in diagnosis of this disease. The OCT, not only can depict the vivid change of foveal schisis, but can also be used for the diagnosis of young children who are generally uncooperative with ERG. Just a single scan of the foveal area is sufficient to detect cystoid changes and to distinguish XLRS from other differential diseases associated with visual loss in uncooperative children^25^,^26^. Importantly, it can be used to detect foveal schisis alone in the eyes with clinical XLRS who show normal ERG results^27^. Rnner *et al.* observed ERG results in the two-year follow-up study and believed more than 40% of patients with RS1 mutations did not have a negative ERG^28^. Thus the appearance of OCT has changed the diagnostic approach for XLRS^28^. Menke *et al.* also found SD-OCT might show absence of retinoschisis in older patients which increases the difficulty to differentiate it from other forms of macular dystrophy^25^ (i.e. II-4). Overall, molecular genetic testis is most precise diagnostic method^26^,^29^.

In summary, we identified a new mutation of XLRS to expand the mutation spectrum and explored the important role of OCT on its diagnosis. Importantly, the inconformity of genotype and phenotype reports deepen our understanding of the molecular basis of XLRS. Finally, OCT proved it is vital in XLRS diagnosis especially in children. A combination of ERG, FAF, OCT, and molecular-genetic testing is advised to verify the diagnosis in clinically suspected XLRS.

## Additional Information

**How to cite this article**: Xiao, Y. *et al.* X-Linked Retinoschisis: Phenotypic Variability In A Chinese Family. *Sci. Rep.* **6**, 20118; doi: 10.1038/srep20118 (2016).

## Figures and Tables

**Table 1:** Pedigree of the family in this study.

No.	Age	Gender	BCVA	Macular Appearance	Peripheral appearance
I-2	81	F	OU 20/120	Normal under ultrasonography	Normal under ultrasonography
II-1	50	M	OU 20/20	NAD	
II-2	48	F	OU 20/20	Nothing especial	
II-3	43	M	OD NLP; OS LP	Vitreous opacity in both eyes, calcification in right eye	
II-4	61	M	OD20/200; OS20/400	Retinoschisis change in right eye; Macular atrophy in left eye by OCT	Pigment epithelium atrophy
II-5	58	F	OU 20/25	NAD	
III-1	27	M	OD 20/20: −2.5 S; OS 20/20: −3.0 S	NAD	
III-2	22	M	OD 20/25:−1.0 S; OS 20/20:−1.0 S	Mild cysts in inner nuclear layer in both eyes by OCT	
III-3	25	F	OD 20/12.5:−1.5 S OS 20/12.5:−1.2 S	NAD	
III-4	34	M	OU 20/16	NAD	
III-5	33	F	OU 20/20	NAD	
III-6	35	M	OU 20/20	NAD	
III-7	30	F	OU20/20	NAD	
III-8	35	M	OD 20/20:−1.0 S; OS 20/20:−1.0 S	NAD	
IV-1	6	M	OD 20/63 : + 2.5 S + 3.0 C 90; OS 20/160: + 4.0 S + 1.5 C 75	Large cyst in multilayer in both eyes by OCT	Schisms
IV-2	4	F		Nothing especial	
IV-3	12	M	OD 20/50: + 2.0 S + 0.5 C 155; OS 20/40: + 2.0 S + 1.25 C 175	Large cyst in multilayer in both eyes by OCT	Schisms and linkage in FA
IV-4	5	M		Schisms in both eyes	

BCVA: best corrected visual acuity; M: male; F: female NAD: No Abnormality Detected S: Sphere; C: cylinder For example OD 20/50: + 2.0 S + 0.5 C at 155 means that best corrected visual acuity of IV-3 was 20/50 with + 2.0 sphere and + 0.5 cylinder at 155 degree in right eye

II-3 had trauma when he was young, and there is lens dislocation and retinal detachment now.
