# Corrigendum: X-Linked Retinoschisis: Phenotypic Variability in a Chinese Family

**DOI:** 10.1038/srep21940

**Published:** 2016-03-10

**Authors:** Yangyan Xiao, Xiao Liu, Luosheng Tang, Xia Wang, Terry G. Coursey, Xiaojian Guo, Zhuo Li

Scientific Reports 6: Article number: 20118; 10.1038/srep20118 published online: 01292016; updated: 03102016

The original version of this Article contained typographical errors in the spelling of the author Terry G. Coursey, which was incorrectly given as Terry Coursy. This has now been corrected in the PDF and HTML versions of the Article.

